# Fabrication of p-type ZnO thin films with high mobility using reactive gases N_2_ and Ar by RF sputtering

**DOI:** 10.1371/journal.pone.0337821

**Published:** 2025-12-17

**Authors:** A. Ismail, Badr Ismael Abdul-Razak, Motahher A. Qaeed, Ammar AL-Farga

**Affiliations:** 1 Department of Physics, University of Hafr Al Batin, Hafar Al-Batin, Saudi Arabia; 2 Chemistry Department, Faculty of Science, Hodeida University, Hodeida, Yemen; 3 Department of Physical Science, Faculty of Science, University of Jeddah, Jeddah, Saudi Arabia; 4 Department of Biochemistry, Faculty of Science, University of Jeddah, Jeddah, Saudi Arabia; Universidad Autónoma de Querétaro: Universidad Autonoma de Queretaro, MEXICO

## Abstract

Co-doping with AlN via RF sputtering is necessary since it is still very difficult to create high conductivity p-type zinc oxide (ZnO) thin films. At room temperature, RF sputtering was used with Ar (20%) and N2 (80%) at a range of target powers (150, 175, 200, 225, and 250 W). All of the produced films displayed the ZnO (002) peak of the wurzite structure. Using the PL approach, the recombination of free excitons was detected. The ZnO:AlN and ZnO:N Raman peaks were observed at 578.58 cm^-1^ and 276 cm^-1^, respectively.With hole concentrations of 3.06 × 10^+16^ cm^-3^ and 1.83 × 10^+18^ cm^-3^, respectively, and corresponding mobilities of 117 cm^2^ V^-1^ s^-1^ and 19.1 cm^2^ V^-1^ s^-1^, the AZO23 and AZO25 samples demonstrated p-type conductivity behavior. The N-Al-N complex, which forms as a shallow acceptor when Zn^+2^ ions are substituted by Al^+3^ ions, is the cause of the p-type behavior of the ZnO sample (AZO23).However, the production of (N)_O_ acceptors due to the substitution of the bigger N^-3^ ions (radius of 0.146 nm) for the O^-2^ ions (radius of 0.140 nm), may be the cause of the p-type behavior of AZO25 sample. AZO23 sample has a greater mobility (117 cm^2^ V^-1^ s^-1^) which can be explained by the higher mean free path/crystallite size (Ɩ/D) ratio.

## 1. Introduction

Despite the exceptional properties of ZnO such as energy gap width up to 3.37 eV and huge exciton binding energy 60 meV, the deployment of ZnO is hindered by the complex process of synthesizing p-type samples [1–4]. the conductivity of n-type play a role of typified of ZnO, which is attributable to its zinc interstitial and oxygen vacancies. Thus, nitrogen (N_2_) is exploited as an approach to p-ZnO doping to enable p-type conductivity. N soluble in ZnO with a low value which initiates at a deep acceptor level. Hence, Yamamto [5] proposed a co-doping approach of elements that contribute charges for the synthesis of p-ZnO with the aim of improving the solubility of N in ZnO to obtain p-ZnO with a higher state of inclusion of N into the ZnO lattice structure. The co-dopant activates the acceptor element, leds to augmenting the density of sites’acceptor and decline in the Madelung energy of delocalized N atoms [5].

Different techniques could be used to synthesize ZnO thin films [6–9]. The RF sputtering technique has attracted enormous interest, as the properties of the ZnO films can be controlled by adjusting the sputtering conditions that include substrate type, annealing temperature, film thickness, RF power, and deposition pressure, as mentioned in previous reports [4,10,11]. This is crucial, as the crystalline, electrical, and optical characteristics of ZnO films depend on these synthesis and deposition conditions. By tuning the Al and N concentrations, p-type doping can be developed.

A literature survey on some electrical properties of ZnO thin films fabricated by RF sputtering is presented and summarized (Table 1). The reported ZnO films exhibited carrier concentration (n or p) in the range (5 × 10^15^–8.68 × 10^20^) cm ^−3^, mobility (μ) in the range (0.1–13 154) cm^2^V ^−1^ s ^−1^ and resistivity (ρ) in the range (4.62 × 10^−4^–97) Ω cm. AlN and ZnO as co-sputtering targets [12], in which Argon only was used as sputtering gas and the as-deposited Al-N codoped ZnO showed n-type conductivity, although nitrogen was detected in the films. Annealing treatment in N_2_ at 400°C-700°C- was carried out for the activation of the nitrogen acceptor, where the films exhibited p-type conductivity with mobility (2.35–6.58) cm^2^V ^−1^ s ^−1^, resistivity (0.524–162) Ω cm and hole concentration (5.04 × 10^18^–5.88 × 10^15^) cm^-3^.

**Table 1:** Electrical properties of RF sputtered ZnO films from selected reports.

#Ref	type	Resistivity (Ω cm)	Mobility (cm^2^ V^-1^s^-1^)	Carrier concentration cm^-3^
[13]	n-type	1.39 *10^−3^	10.11	5.35 *10^20^
[14]	n-type	4.4810^−4^	47	
[15]	n-type	5 × 10^−3^	4-18	3 *10^20^
[16]	p-type	3.5	1.2	10^18^
[17]	p-type	7.73	0.86	9.36*10^17^
[18]	p-type n-type	0.5 2.36	1.2 1.1	2.4 *10^18^ 1.73 *10^19^
[19]	p-type	2.6	9.6	2.5 *10^17^
[20]	p-type	3.21	0.09	2.11*10^19^
[21]	n-type	0.1	0.85	1*10^20^
[22]	p-type	10.4 - 19.3	4- 13	2.7*10^16^ – 2.2*10^17^
[23]	n-type	2*10^−4^	38.9	6*10^20^
[24]	p-type	1.97*10^−2^	12.36	2.56*10^19^
[25]	p-type	0.0134	154	3.03*10^18^
[26]	n-type	4.62*10^−4^	15.6	8.68*10^20^
[27]	p-type	–	0.1	8*10^17^
[12]	p-type	30	64	5*10^15^
[28]	p-type	0.524- 162	2.35-6.58	5.04*10^18^ – 5.88*10^15^
[29]	p-type	97.9	6.7-2.3	3.4*10^16^ – 1.2*10^16^

Thus, the present work by, using N_2_ and Ar as sputtering gases, aims to produce high mobility P-type ZnO under different fabrication conditions. The mechanism for the formation of p-type ZnO is discussed based on the structural, morphological, electrical and optical characteristics of the films obtained. The main objectives of the present work are as follows:

- To synthesize p-type of Al-N codoped ZnO films with higher mobility using RF sputtering technique
- To investigate the effect of fabrication conditions (RF power, sputtering gases,) on the structural, morphological, electrical and optical characteristics of the films
- To elucidate the mechanism for the formation of p-type ZnO film based on the measured films

## 2. Experimental

“All data generated or analyzed during this study are included in this published article”

In this work, RF sputtering technique utilized to synthesize ZnO thin films doped with AlN deposited on n-type-Si (100) and glasses substrates. The substrates were washed in an ultrasonic bath that contains acetone and isopropanol, which were then rinsed with de-ionized water. A500 RF sputtering equipment was utilized to fabricate the thin films under the deposition conditions presented in Table 2.

**Table 2:** Deposition conditions of AlN: ZnO films.

Sample	Target Power	Working gas (Ar:N_2_)	Substrate temperature	Working pressure (mbar)
AZO22	ZnO (150 W) AlN (100 W)	Ar (20%) N_2_ (80%)	RT	2 × 10^−2^
AZO23	ZnO (175 W) AlN (100 W)	Ar (20%) N_2_ (80%)	RT	2 × 10^−2^
AZO24	ZnO (200 W) AlN (100 W)	Ar (20%) N_2_ (80%)	RT	2 × 10^−2^
AZO25	ZnO (225 W) AlN (100 W)	Ar (20%) N_2_ (80%)	RT	2 × 10^−2^

Raman spectroscopy, photoluminescence (PL) and UV-visible spectrophotometry were used to inspect the optical properties of the samples. The different thicknesses (t) of the prepared films were determined via F20 filmetrics device. X-ray diffractometer (source Cu K_α_ with λ = 1.5406 Å) and field emission scanning electron microscopy (FESEM) were utilized to explore the crystalline structure and surface morphologies of the fabricated thin films, respectively. Hall Effect equipment (HL5500PC model) was utilized to examine electrical properties, particularly the type of the prepared samples.

## 3. Results and discussion

**Fig 1:**
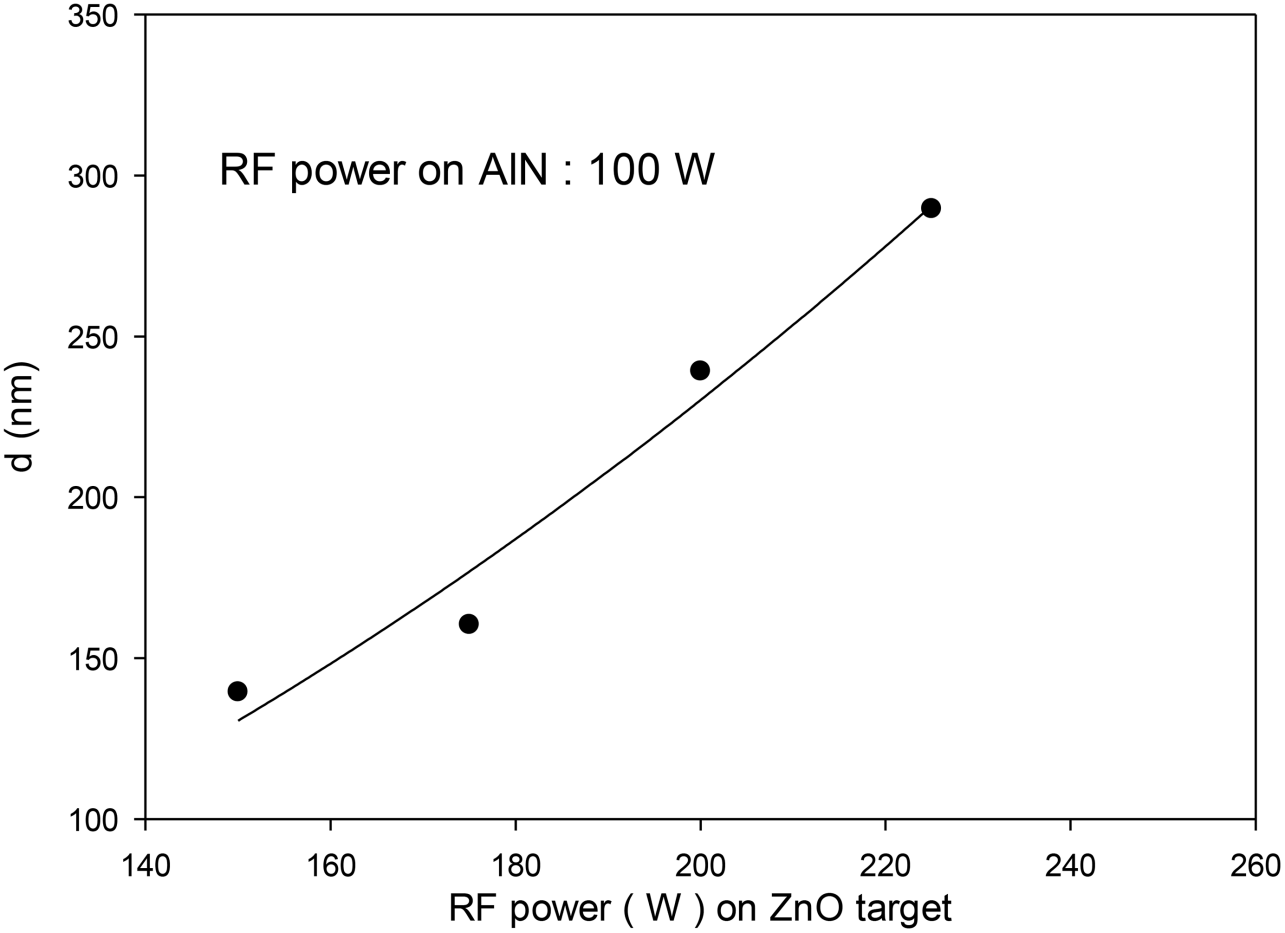
Thickness of the films as a function of the RF power on ZnO target and 100 W on AlN target.

As observed, the film thicknesses (d) varied from 139.4 nm to 290 nm with increase in power of RF applied on ZnO target from 150 W to 225 W. Thus, it can be inferred that there is a direct proportionality between the number of sputtered atoms and applied RF power (Fig 1).

**Fig 2:**
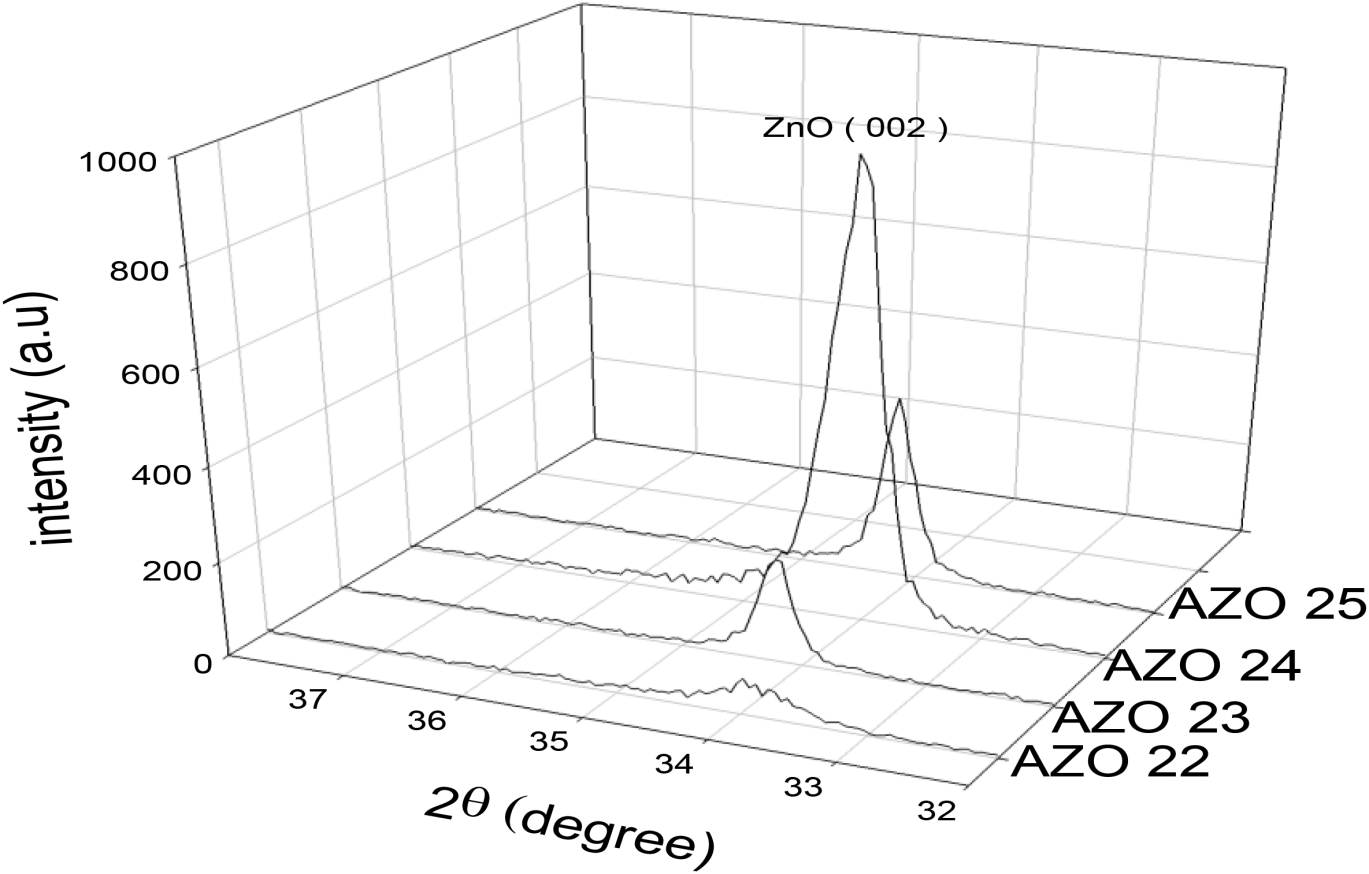
XRD spectra of AlN: ZnO thin films.


The Xray diffractogram of ZnO films doped with AlN are presented in Fig 2. The (002) ZnO orientation appeared in all the films with the highest intensity exhibited by the AZO 24 sample. Also, by comparatively analyzing the diffraction spectra obtained for the doped thin films against that of the bare ZnO presented in an earlier study [10], the two theta value and interspacing (d) of the ZnO were found to be equal to 34.1841º and 2.6229Å, respectively [10]. The least d-spacing value of 0.26199 nm of the AZO23 sample resulted due to the substitution of Al^+3^ ion, which has a radius of 0.53 Å with Zn^+2^ ion, which has a larger radius of 0.74 Å, leading to a shift in (002) peak position to a larger value. However, the d-spacing values for AZO 22, AZO 24 and AZO 25 surpasses that of the bare ZnO, possibly as a result of the replacement of O^-2^ ions, which has a radius of 1.40 Å with N^-3^ ions that has a larger radius of 1.46 Å, resulting in the shift of (002) to a smaller angle. Using the Scherrer’s formula [Eq. 1], the X-ray variables and FWHM of ZnO (002) peak were input to determine the crystallite size (D) of the samples.

$$D=\frac{\text{0}\text{9}\text{4}\lambda }{\beta cos\theta }$$ (1)

where *λ is* X-ray wavelength*, θ* is the Bragg angle, and *β* is full width at half maximum (FWHM). The crystallinity of the prepared samples, AZO 22 to AZO 25, has improved from 14.7 nm to 29.4 nm due to the diffraction peaks becoming sharper and shifting slightly toward higher 2θ values. The lattice parameters come out from XRD spectra of ZnO thin films doped with AlN (002) are shown in Table 3.

**Table 3:** XRD results of AlN: ZnO thin films.

Sample	2 ɵ (˚)	FWHM(˚)	d (Å)	c (Å)	D(nm)
AZO 22	33.9036	0.5904	2.6441	5.2882	14.70
AZO 23	34.1750	0.4000	2.6199	5.2399	21.71
AZO 24	34.0410	0.3444	2.6338	5.2675	25.21
AZO 25	34.1799	0.2952	2.6234	5.2467	29.42

Table 4 presents the EDX elemental composition of ZnO films doped with AlN wherein trace amounts of nitrogen (0.10–1.26%) are observed. Despite fixing the power at 100 W of RF sputtering on AlN, it is imperative to note that the concentrations of N and Al were varied. These variations in concentrations of incorporated N and Al were achieved by applying various RF powers on ZnO target and the inclusion of N in the course of sample preparation and synthesis, leading to the generation of (N)_O_ acceptors or (N_**2**_)_O_ donors. Another plausible mechanism is the production of N-Al-N complex pattern N_O_ (N on O) as well Al_Zn_ (Al on Zn), which boosts strong interactions between acceptor (N) and donor (Al), stabilizes ionic charges and limits the acceptors interacts. Also, the acceptor and donor’s binding energies can be reduced and increased, respectively, thereby the solubility of N-Al-N compounds in the AlN doped ZnO films will be improved [5].

**Table 4:** EDX analysis of AlN: ZnO films at different RF powers on Si substrates.

Sample	NK (at %)	O K (at %)	Al K (at %)	Zn L (at %)
AZO22	0.48	54.13	3.42	41.98
AZO23	1.26	53.34	2.51	42.89
AZO24	0.56	51.42	1.48	46.53
AZO25	0.10	51.38	0.60	47.97

**Fig 3:**
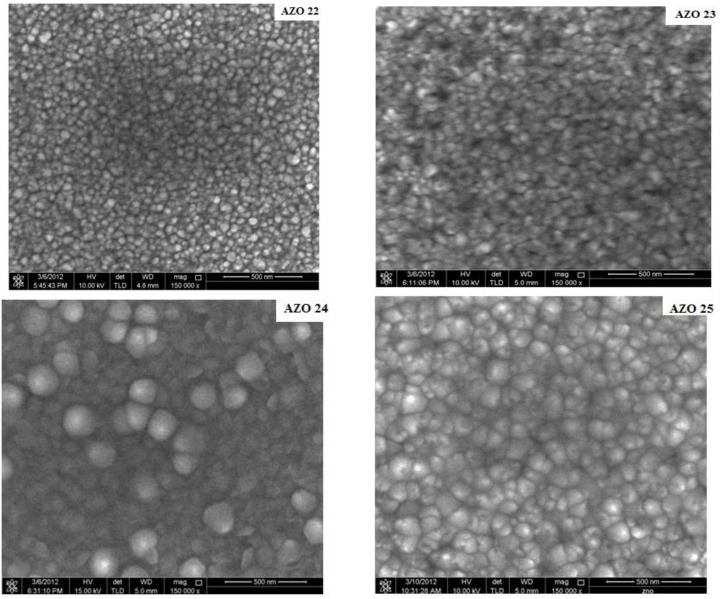
FE-SEM images of AlN: ZnO thin films.

**Fig 4:**
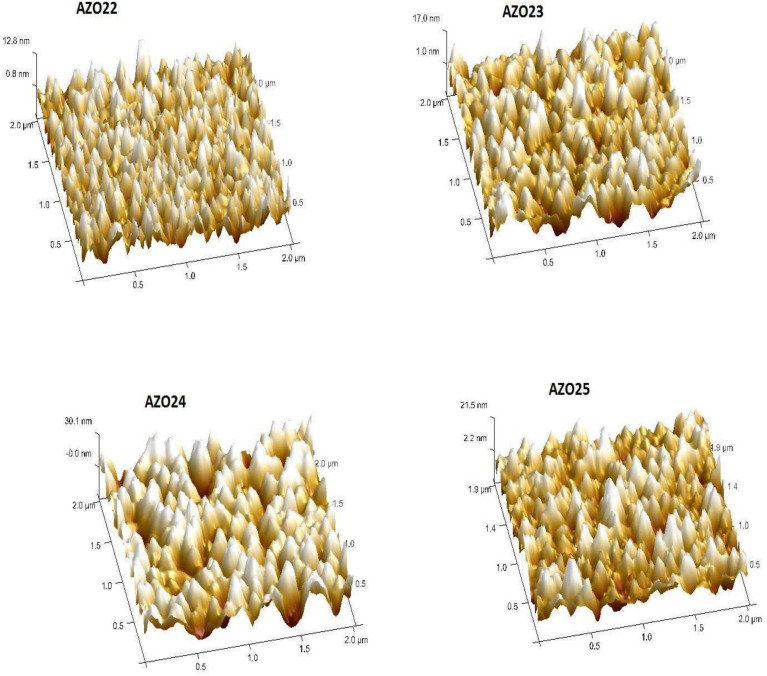
AFM result of AlN doped ZnO thin films.

As shown in Figs 3 and 4, AFM and FESEM were employed to investigate the doped films microtopography and morphological variations. The root mean square (rms) for AZO22, AZO23, AZO24 and AZO25 were 3.05 nm, 3.87 nm, 6.60 nm and 4.89 nm, respectively occure with increasing RF power on ZnO target. Also, the AFM images show that the surface morphology and roughness of AZO thin films (AZO 22–25) clearly vary with changes in growing circumstances. AZO 22 shows small grains with a smooth surface and a maximum height of ~12.8 nm in comparison with AZO 23, which shows larger surface characteristics with higher peak heights of ~17.0 nm, indicating the onset of grain coarsening. In AZO 24, the surface roughness dramatically increases to 30.1 nm, indicating noticeable surface imperfections and grain development. Finally, AZO 25 has a rougher surface with peak heights of about 21.5 nm and more evenly spaced features.

Furthermore, it is discernible from the FESEM results that the grain size of the films was enhanced with increasing RF power on ZnO target, which is compatible with the XRD data. The small and densely packed grains in the AZO 22 nucleation-dominated stage show that limited adatom mobility restricts grain formation. For AZO23, the grains seem more linked, which may indicate that adatoms became more mobile and that smaller crystallites were able to combine to form larger grains. In AZO24 and AZO25, this pattern is particularly noticeable, where noticeable grain coarsening is seen in the emergence of distinct, almost spherical grains. By decreasing the overall grain border area, the system naturally minimizes surface energy, which leads to such grain growth. As noted in earlier studies, the number of sputtered molecules that arrive at the substrate surface is the prevailing factor that controls the morphology of the films [21,30].

UV-vis transmission spectrometer was utilized to calculate the energy band gap (Eg) of the samples. The photon energy (E) and the absorption coefficients α are given by Eq. (2) below;


$$\begin{array}{c} \alpha E=A(E-E_{g}{)}^{\frac{\text{1}}{\text{2}}},\;\alpha =\frac{\text{1}}{t}Ln\left(\frac{\text{1}}{T_{i}}\right) \end{array}$$ (2)

where A is a constant and t is the film thickness. Therefore, the plot of energy E against (αE)^2^ showed a linear line that cutting of the energy axis at the E_g_ value.

**Fig 5:**
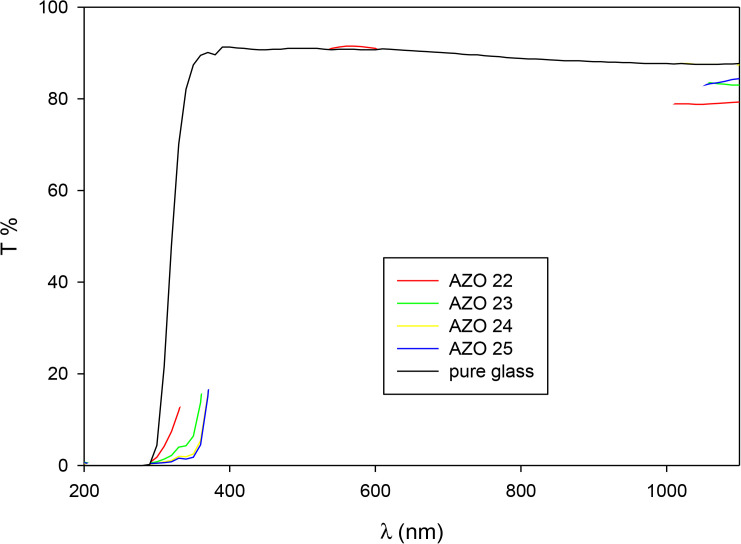
Transmittance spectra of the prepared thin films.

**Fig 6:**
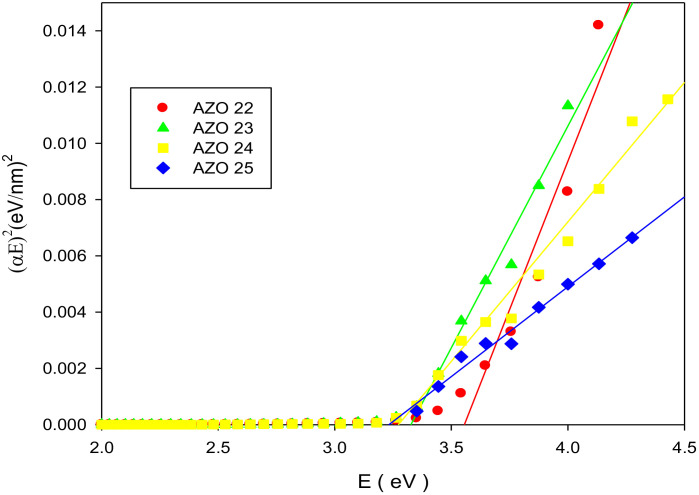
(αE)^2^ versus E of AlN: ZnO films on glass at various RF powers on glass substrates.

The prepared films showed an optical transmittance that exceeds 76% in visible range, as shown in Fig 5. Plot of (αE)^2^ against E yielded band gap of 3.56, 3.33, 3.28 and 3.24 eV occurring with RF powers of 150, 175, 200 and 225 W respectively, as presented in Fig 6. The lowering in energy band gap from 3.56 eV to 3.24 eV could be related to the N doping exceeding the valence-band, irrespective of the ZnO films’ conduction type [19].

**Fig 7:**
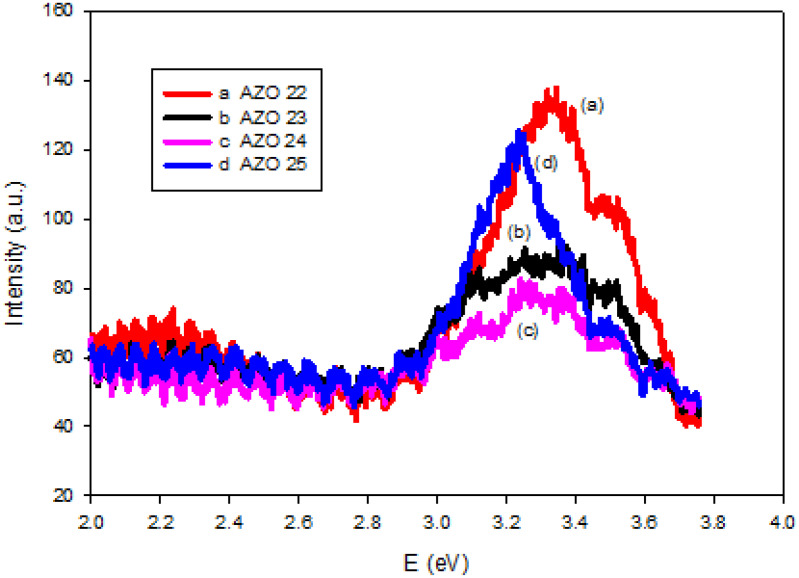
The PL spectra of AlN: ZnO thin films at different RF powers on Si substrates.

The photoluminescence (PL) spectra of the thin films (Fig 7) were acquired under ambient conditions. The characteristic UV emission peaks of free exciton recombination are found in all prepared films, indicating good optical properties and good crystalline structure [10,31]. The relatively low intensity of broad visible emission in the synthesized ZnO samples compared to undoped ZnO indicates a decline in the intrinsic defects effect and the successful doping of ZnO films with AlN [10, 32,33]. With increasing Al concentration from 0.6% to 3.42%, there is an observed shift in UV emission band from 3.24 eV to 3.33 eV as a result of the Moss-Burstein effect, in accordance with the transmittance data.

**Fig 8:**
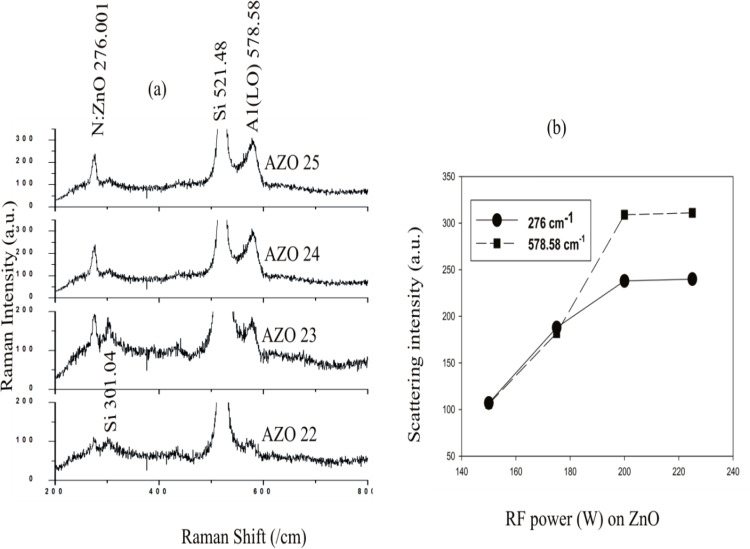
(a) Raman spectra of the thin films and (b) modes intensity variation at different.

Raman spectra for the prepared films is shown in Fig 8 (a). The peak at 276 cm^-1^ denotes nitrogen ^18^. Compared with the undoped ZnO, there is a discernible shift in the peak denoting A1 (LO) modes [30] at 573.72 cm^-1^ to 578.58 cm^-1^ for the sample synthesized, indicating the successful doping of the thin films with AlN [34, 35]. There is a linear correlation of the intensities of Raman modes at 57858 cm^-1^ and 276 cm^-1^ with N concentrations in the films, which can be utilized as an important parameter for calculating the relative concentration of N in the films [36]. As well as the modes intensity depends on the applied RF power to the ZnO target, as it shown clearly in Fig 7 (b). The ZnO samples prepared via RF powers at 200 W and 225 W exhibited higher N concentrations as compared to the samples prepared at 150 W and 175 W. This is clearly observed in Fig 8 (b), where these samples show higher mode intensities at 276 cm^-1^ and 578.58 cm^-1^ compared with those samples prepared by ZnO target with RF power of (150 W and 175 W). Besides N concentration, the conductivity type of the films is dependent on the N_2_ (molecular): N (atomic) ratio, as reported in a previous study [20], and further shown in the analysis of electrical properties of the synthesized samples. Peaks at 301.04 cm^-1^ and 521.48 cm^-1^ (Fig 8-a) denote the Si substrates [37].

**Fig 9:**
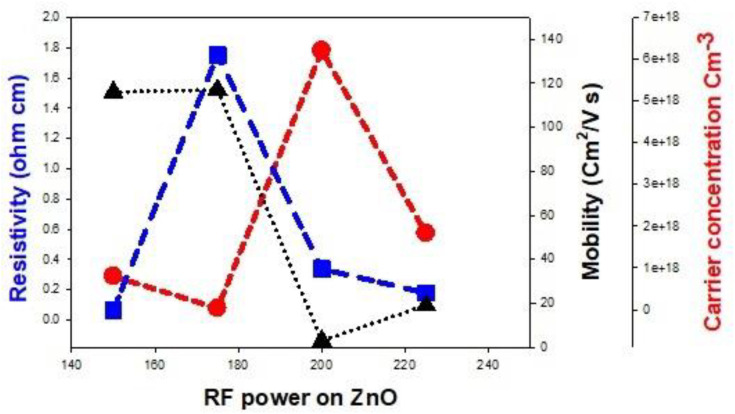
The electrical properties of the prepared thin films.


Table 5 and Fig 9 show the electrical properties derived from Hall effect measurement. It is well known that O vacancies (V_O_), Zn interstitial (Zn_i_), substitutional Zn on O site (Zn_O_), N interstitial (N_i_), N_2_ on O site (N_2_)_O_ and Al on Zn site (Al_Zn_) are donors while N on O site (N_O_), O interstitial (O_i_) and Zn vacancy (V_zn_) are acceptors. The results of mobility, carrier concentration and resistivity were shown in Table 4. The samples (AZO 23 and AZO 25) display p-type conductivity with hole concentrations of 3.06 × 10^+16^ cm^-3^ and 1.83 × 10^+18^ cm^-3^ respectively. This p-type conductivity behavior of AZO 23 sample results from production of N-Al-N pattern that serve as a shallow acceptor, which results from the substitute of Zn^+2^ ions with Al^+3^ ions. This result is appropriate with the XRD result, EDX and Raman investigations, where the AIN doped ZnO sample showed smaller interface (d) of 0.26199 nm in comparison with a bare ZnO (0.26229 nm) in addition to the nitrogen detected in these samples. The behavior of p-type of AZO 25 sample is because of the compensated of O^-2^ ions (radius of 0.140 nm) in N^-3^ ions (radius of 0.146 nm) and the generation of (N)_O_ acceptors and (N_2_)_O_ donors. The effect of (N_2_)_O_ donors is smaller than that of (N)_O_ acceptors resulting in p-type conductivity in this sample. This deduction is consistent with the compositional and structural data obtained from XRD, Raman, and EDX results, where N was detected in the synthesized samples, and the AIN doped sample showed larger d-spacing values of 0.26234, 0.26441 and 0.26338 nm for AZO 22 and AZO 24 samples as compared with 0.26229 nm for undoped ZnO. AZO 22, AZO23 and AZO 24 samples also displayed n-type conductivity with concentrations of 8.00 × 10^+17^ cm^-3^ and 6.20 × 10^+18^ cm^-3^. This implies that N^-3^ successfully replaced O^-2^ ions; By any way, the impact of (N)_O_ acceptors is smaller than that of (N_2_) _O_ donors and the other intrinsic donors, appearing in n-type conductivity of these samples. The variability in mobility for the different samples may arise from the nature of the grain – boundaries in the samples. To calculate the contribution of the grain – boundaries, the mean free paths of the carriers in the films were calculated using Eq. 3 below:

**Table 5:** The electrical properties of the prepared thin films.

Sample	Thickness, t (nm)	RF power on ZnO (W)	Resistivity (Ω_-_cm)	Mobility (cm^2^ V^-1^s^-1^)	Carrier concentration (cm^-3^)
AZO22	139.4	150	0.067	116	−8 × 10^ + 17^
AZO23	160	175	1.75	117	3.06 × 10^ + 16^
AZO24	239.2	200	0.337	2.99	−6.20 × 10^ + 18^
AZO25	290	225	0.179	19.1	1.83 × 10^ + 18^

$$\begin{array}{c} \text{l}=\frac{\text{h}}{\text{2e}}\times {\left[\frac{\text{3n}}{\pi }\right]}^{\left(\frac{\text{1}}{\text{3}}\right)}\times \mu \end{array}$$ (3)

where Ɩ, n and µ represent mean free path, concentration of carriers, and mobility, respectively. The mean free paths were calculated to be 2.19 nm, 0.75 nm, 0.11 nm and 0.47 nm for AZO22, AZO23, AZO24 and AZO25, films respectively. Also, the ratio of the mean free path to the crystallite size (Ɩ/D) were found to be 16%, 3%, 0.44% and 1.5% for the respective films, which are higher than earlier reported values (e.g., 0.12%). The higher Ɩ/D ratios for AZO22 and AZO23 films compared with AZO24 and AZO25 films contributed to the higher mobility of carriers in the former films (AZO22 and AZO23).

## 4. Conclusion

This study synthesized p-type ZnO thin films doped with AlN via RF magnetron sputtering technique at different RF powers (150, 175, 200, 225 and 250 W). All the films displayed a wurtzite structure that is preferentially oriented along the (002) plane. The direct proportionality between number of sputtered atoms and applied RF power is indicated by the increase in film thickness with RF power. With increasing the power of RF sputtering on ZnO target, the structure and grain size of the AIN doped films were enhanced. The decline in optical energy gap from 3.56 eV to 3.24 eV with increasing RF power can be attributed to the N doping above the balance-band. The relatively low intensity of broad visible emissions in the prepared ZnO samples indicates a decline in the impact of intrinsic defects and the successful doping of ZnO films with AlN.

The co-doping of ZnO with AlN enabled p-type conductivity and increased the mobility of ZnO. The samples doped at RF power of 175 and 225 W show p-type conductivity with mobilities of 117 cm^2^ V^-1^ s^-1^and 19.1 cm^2^ V^-1^ s^-1^, with corresponding hole concentrations of 3.06 × 10^+16^ cm^-3^ and 1.83 × 10^+18^ cm^-3^ respectively. The behavior in p-type sample doped at 175 W of RF power is attributable to the generation of N-Al-N pattern that has appeared as a shallow acceptor, which results from the replacement of Zn^+2^ ions with Al^+3^ ions. On the other hand, the p-type behavior of the sample doped at RF power of 225 W is ascribed to replacement of O^-2^ ions (radius of 0.140 nm) with the larger N^-3^ ions (radius of 0.146 nm) in turn led to formation (N)_O_ acceptors and (N_2_)_O_ donors. This also explains the variability in mobility values. The impact of (N_2_)_O_ donors is smaller than that of (N)_O_ acceptors, which accounts for the p-type conductivity. Moreover, the higher mean free path/crystallite size (Ɩ/D) ratio recorded for the sample doped at 175W accounts for its higher mobility (117 cm^2^ V^-1^ s^-1^).
